# E, K, B5, B6, and B9 vitamins and their specific immunological effects evaluated by flow cytometry

**DOI:** 10.3389/fmed.2022.1089476

**Published:** 2023-01-05

**Authors:** Camelia Munteanu, Ioana Berindean, Mihaela Mihai, Bianca Pop, Mihai Popa, Leon Muntean, Olivia Petrescu, Andreea Ona

**Affiliations:** ^1^Department of Plant Culture, Faculty of Agriculture, University of Agricultural Sciences and Veterinary Medicine, Cluj-Napoca, Romania; ^2^Department of Transversal Competencies, University of Agricultural Sciences and Veterinary Medicine, Cluj-Napoca, Romania; ^3^Department of Applied Modern Languages, Faculty of Letters, Babeş-Bolyai University, Cluj-Napoca, Romania

**Keywords:** anticancer, diseases, flow cytometry, immunology, vitamins

## Abstract

It has been proven that vitamins play an essential role in preventing certain diseases since ancient times. It is thus fruitless to approach the roles of vitamins without making reference to the techniques used in evaluating the effects of these micronutrients. Therefore, the aim of this paper was to summarize the immunological effects of E, K, B5, B6, and B9 vitamins evaluated by flow cytometry. Some of these significant effects were presented and discussed: (a) The role of vitamins E in the prevention and treatment of different types of cancer. (b) The properties of K vitamins in the development and maintenance of pheochromocytoma Cell Line 12 (PC12) cells in Parkinson’s disease; (c) The improvement effect of vitamin B5 on the loss of bone mass in low estrogen conditions; (d) The anticancer role of vitamins B6. (e) The role of Vitamin B9 in the regulation of Treg cells. As such, the flow cytometry technique used to assess these properties is essential to evaluate the immunomodulatory effects of certain vitamins. The technique undergoes constant improvement which makes it possible to determine several parameters with a role in the modulation of the immune function and at the same time increase the accuracy of the methods that highlight them.

## 1. Introduction

Considering that the spread of diseases has always been a public health problem, the use of prevention methods was approached in this respect (1). Vitamin administration is one such method (2). The term comprises two words, vital and amine, even though it was later demonstrated that not all vitamins are amines (3). In this regard, vitamins are vital for maintaining homeostasis (4). Certainly, their health effects highly depend on the doses. There are 13 vitamins that are divided into fat and water soluble (5). As for E and K fat-soluble vitamins, they are absorbed (6) in the small intestine, especially in the distal part, in the presence of lipids. After absorption, they may easily be stored. In contrast, the absorption of water-soluble vitamins occurs in different ways. With the exception of B9, B7, and B2, all water-soluble vitamins are absorbed only in the small intestine (7). For the rest, the absorption involves large intestine microbiota (8). For instance, an essential antioxidant for biological membranes, vitamin E (RRR) has several stereoisomers, with RRR-α-tocopherol being the most abundant (9). It has anticancer effects *via* its anti-inflammatory mechanisms (10). Vitamin K is another vitamin with anticancer effects *via* the modulation of some transcription factors such as Fos and Myc, which are implied in the progression and proliferation of tumor cells (11). It is considered an agent that increases insulin sensitivity and is involved in the bone formation process (12). Vitamin B5 (VB5) is a precursor for CoA, and thus it is responsible for many biochemical and signaling reactions in the human body such as β-alanine biosynthesis, CoA metabolism, and regulation of insulin release (13–15). There are cases when flow cytometry was used to assess changes during VB5 administration. In this respect, it revealed the shape change of platelets in platelet-rich plasma (PRP) in terms of its antithrombotic properties (15). Moreover, some chronic diseases are suppressed by vitamin B6, which is widely used due to its anti-inflammatory properties (16, 17). Moreover, regulatory T-cells need to survive vitamin B9 because these cells are capable of highly expressed B9 folate receptors 4 (FR4) on their surface. T-cells differentiate from naïve T-cells expressed on their surface FR4 and need B9 to survive. The absence or insufficient quantity of B9 causes T-cell apoptosis. In addition, anti-apoptotic Bcl-2 expression is decreased and intestinal inflammation increases. This requirement was confirmed by the use of flow cytometry (18).

The roles of vitamins cannot be approached without significant reference to the techniques used in the evaluation of the effects exerted by these micronutrients. Moreover, flow cytometry was chosen by many researchers to highlight vitamin properties as a reliable technique that is used in a variety of domains like immunology, hematology, cancer biology, etc., as a method to assess the immune response to treatment (19).

## 2. Flow cytometry (FC) applications

Due to the wide range of methods that involve FC, and the variety of antibodies that can bind specific proteins from different cellular compartments, the field of FC is continuously developing and these methods can be applied in biomedical investigations. Regarding immunology applications, flow cytometry is widely used for immunophenotyping. Also, it is applied to intracellular cytokine proliferation, apoptosis, and cell cycle analyses (19).

On the one hand, the technique used is based on the measurement of DNA content in certain phases of the cell cycle in a population (cell cycle analysis). Thus, it was observed that the amount of DNA increases progressively from G1 to G2. Moreover, in G2, it reaches double compared to G1 and G0 (resting cells). The advantage of the specific use of fluorochromes in this technique made the total estimation of the DNA quantity in the cells possible (20). On the other hand, regarding cancer cells, flow cytometry was used to evaluate the inhibitory effect on the cell cycle by vitamins K1, K2, and K3 through the DNA content of PLC/PRF/5 human HCC cells that were treated with them. 3-(4,5-Dimethylthiazol-2-yl)-2,5-diphenyltetrazolium bromide (MTT) analysis revealed that the PL/PRF/5 cells could double in about 48 h. To ensure that the cells were synchronized at the G0 phase of the cell cycle, they were treated with and without K1, K2, and K3 at 90 μm concentration because this concentration of vitamin K2 and K3 inhibited the growth of PLC/PRF/5 human HCC cells (21). The growth and maintenance of PC12 cells under vitamin K2 treatment were evaluated by flow cytometry (22). The flow cytometry and PI staining techniques were used to determine the number of apoptotic cells that were produced by PC12 cells (apoptosis analysis). These cells were grown and differentiated using the PI compound and NGF in 12-well plates for 2 days. As such, while the effects of 6-hydroxydopamine (6-OHDA) on the maintenance of cell viability were not significant, the protection against pheochromocytoma Cell Line 12 (PC12) was observed in the presence of vitamin K2 (23). Also, vitamin K2 is involved in the maintenance of cell viability through inhibited activation of the apoptosis-inducing factor in the cell. In this case, flow cytometry was performed to analyze cell differentiation. The expressions of CD14 and CD11b were analyzed for their role in the differentiation of CD34-positive cells (immunophenotyping). The cells were washed with PBS and then incubated for 30 min on ice, on a combination of FITC conjugated anti-CD14 MoAb (Immunotech, Marseilles, France) PE-conjugated anti-CD14 MoAb (Becton Dickinson Biosciences, San Jose, CA, USA). The expression of the transferrin receptor (TfR) and the glycoprotein A (GPA) on ECFCs was analyzed for the differentiation of erythroid cells. The cells were then treated with the combination that was used for CD34-positive, using FITC-conjugated anti-GPA MoAb and PE-conjugated anti-CD71 MoAb (both from Immunotech). Samples were analyzed using a FACSCalibur (BD Biosciences) (24). In patients with different types of leukemia vitamin K2 can have an apoptosis-inducing effect on their newly isolated cells. This is done through a multi-color flow cytometry analysis that uses the antibody known as APO2.7. The mitochondrial antigen expressed by cells undergoing apoptosis, 7A6, is detected by this technique. The results of the study showed that the presence of CD95 (anti-Fas) in the apoptotic Jurkat cells was restricted using a combination of mAb (APO2.7) and non-anti-Fas. The presence of the APO2.7-positive cells was consistently detected by flow cytometry when the concentrations of the CTB-1 suspension were at least 5% (25).

Moreover, in the case of T-cells that express FR4 on their surface and need B9 to survive, flow cytometry was used to distinguish between dead and living cells. The cells were stained with CD4-specific fluorescent antibodies after they were pre-incubated with anti-CD16/32 antibodies. To discriminate between dead and living cells, a *Via*-probe solution (BD Biosciences) was used. Foxp3 (eBioscience, San Diego, CA, USA), phosphorylated STAT5, Ki67, and Bcl2 (BD Biosciences) were stained intracellularly according to the manufacturer’s instructions. Flow cytometry was performed using FACSCanto II and FACSAria systems (BD Biosciences), respectively. As such, with the help of this technique, the requirement of vitamin B9 for the survival of Treg cells was observed (18). In addition, flow cytometry can be a technique used in regenerative medicine. In this case, flow cytometry was used to highlight that CD34+ cells, present in the bone marrow, can differentiate into pancreatic β-cells, thereby showing that they possess pancreatic endocrine potential (immunophenotyping) (26). Interestingly, some authors used flow cytometry to evaluate the effect of natural teeth whitening compounds compared to conventional treatment (27). Therefore, the aim of this paper is to summarize the immunological effects of E, K, B5, B6, and B9 vitamins which were evaluated by flow cytometry.

## 3. Fat-soluble vitamins E and K

Fat-soluble vitamins are necessary to maintain good health, ensure adequate growth and development of the body, and support vital functions. Vitamins A, D, E, and K are considered lipo-soluble vitamins because they have a similar mode of fat absorption and transport (28). Of the 4, E and K will be the main focus. Fat-soluble vitamins are absorbed by the body in the small intestine in the form of micelles. Lipid clusters contain hydrophobic portions inside and hydrophilic portions outside of the micelles. The process takes place with the help of pancreatic enzymes and bile. After being absorbed in the small intestine, the fat-soluble vitamins are packaged in chylomicrons which are taken over by the lymphatic system before reaching the blood. Chylomicrons are metabolized by lipoprotein lipase, which causes the release of fat-soluble vitamins into the tissues for use and storage. Consequently, protein lipase from the pancreatic juice cleaves chylomicrons, resulting in fat-soluble vitamins that are distributed to the tissue, where they can be used for storage (29).

### 3.1. Vitamin E

**FIGURE 1:**
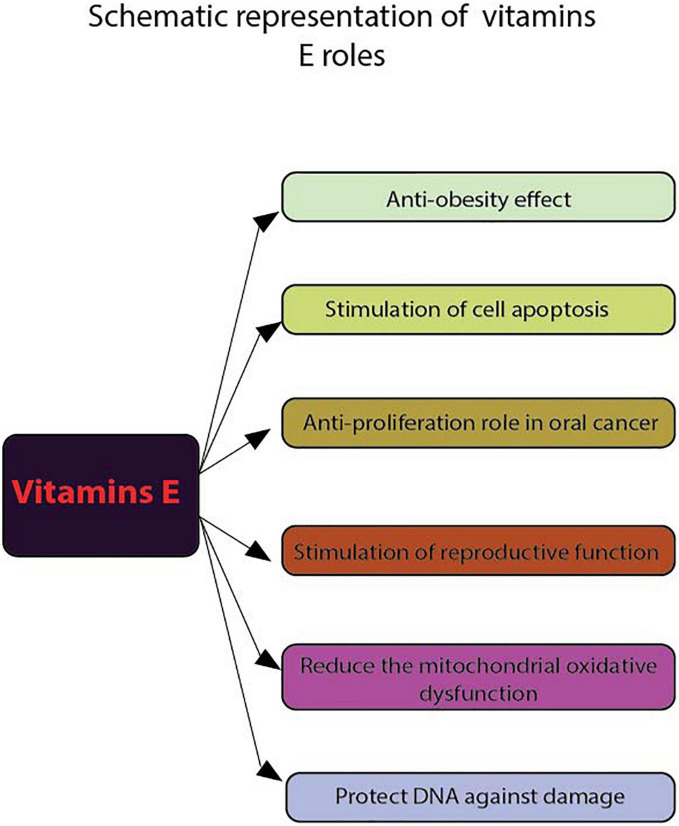
Schematic representation of vitamins E roles. Using flow cytometry, the following functions of vitamins E were highlighted: anti-obesity effect, stimulate cell apoptosis, anti-proliferation role in cancer, stimulate reproductive function, reduce the mitochondrial oxidative dysfunction and protect DNA against damage.

Vitamins E is the collective name of a group of eight different compounds α-, β-, γ-, and δ-tocopherols and the corresponding four tocotrienols (30). One of these, α-tocopherol, is the most abundant and active form in humans and is accepted as a major free radical that eliminates antioxidants and protects biological molecules from detrimental oxidative modifications (31) and lipid peroxyl radicals. The tocopherols can trap propagating radical intermediates that are produced during lipid peroxidation and arrest chain reactions (32). In contrast with vitamin B, vitamin E is insoluble in water, but it is a fat-soluble vitamin and can solely be synthesized in plastids. All tocopherols are found in large quantities in leaves, buds, seeds in the germination state, but also in plant and vegetable oils, therefore only photosynthetic organisms are able to synthesize vitamin E (30). Moreover, vitamin E has multiple applications, as it is widely used as dietary supplement alongside other micronutrients, such as vitamin C and contributes to reducing the risk or preventing diseases that occur due to oxidative changes in biological molecules. Many studies confirm that vitamin E has been found to have beneficial effects for animals (murine), humans and non-murine animals. Studies were developed on animal models, like mice, or cell lines to study various diseases. The latest study on obesity by Kato et al. (33) was conducted on mice fed with a high-fat diet and concluded that tocotrienols (T3s) exerted an anti-obesity effect, normalizing body mass, serum cholesterol and white adipose tissue (Figure 1). It suppressed liver damage, but no changes in cognitive dysfunction were detected. Furthermore, vitamin E mitigated the adverse effect on reproductive performance in mice treated with TCDD (2,3,7,8-tetrachlorodibenzo-p-dioxin) (34) and it can inhibit the oxidative stress induced by some heavy metals such as arsenic, cadmium, and alcohol. In this way, it considerably reduces the harmful effects on the functions of the reproductive system (spermatogenesis) (35). When the rats were treated with different doses of vitamin E over a period of 30 days, their kidneys and livers were affected, but proved it has no adverse effect on the testes. As such, El-Hak et al. (36) recommended exercising caution when taking high doses of vitamin E. Biological and histological modifications that appear due to exposure of mice to CPF (chlorpyrifos) during gestation and lactation were reversed by an oral supplementation of vitamin E, either to the dams or to their pups (37).

Regarding human studies, in a group of employees working on extremely low frequency electromagnetic fields (ELF-EMFs) and treated with vitamin E and C, the results show that these antioxidants can increase the activity of the non-enzymatic antioxidant defense system and protect DNA from damage (Figure 1). To evaluate cell death induced by DNA damage Hosseinabadi et al. (38) used Annexin V FITC/P.I. flow cytometry assay to assess early/late apoptosis and necrosis induced by external factors. Apparently, receiving these vitamins affect the viability of the investigated cells.

Also, vitamin E is being studied in the prevention and treatment of different types of cancer. The use of flow cytometry (FACscanto II system) and FITC Annexin V kit (39) has demonstrated the antiproliferative effect of α-tocopherol on oral cancer cells ORL-48 at a low dose and vitamin E has also been reported to induce apoptosis in erythroid leukemia and in prostate and breast cancer cells (40) (Figure 1).

The same technique was used for non-murine animal studies with FACSca, BD Biosciences (San Jose, CA, USA) (41) to determine the lymphocyte proliferation and peripheral blood mononuclear cell (PBMC) in 15 healthy dogs fed with different types of diet supplemented with either sunflower oil, menhaden fish oil and α-tocopherol acetate for 12 weeks. The results show that the high plasma levels of vitamin E appear to inhibit this suppression of the lymphoproliferative response. Also, the result of flow cytometric measurements (PAS III, Partec GmbH, D-Munster) indicate that vitamin E exerts significant protecting effects on metabolic processes reflected by intracellular glutathione, pH and cellular viability after UVB irradiation (42). On the other hand, even though it is not yet clear how vitamin E reaches the mitochondrial membranes in cells, it is evident that its administration can reduce mitochondrial oxidative dysfunction (32).

Hu and his team (43) proved that GPX4 and vitamin E cooperatively maintain lipid redox balance and prevent ferroptosis in HSPCs, while O’Brien et al. (44), used flow cytometry (FACSCalibur and CellQuest software–Becton Dickinson Instruments, Cambridge, MA, USA) to investigate the effect of dietary supplementation with Vit E and/or Se on immunological parameters in cats. The results show that vitamin E supplementation significantly increased the phagocytic activity and lymphocyte proliferation at a moderate dose (225 mg/kg DM) and did not alter the percentage expression of cell surface markers. This dose appears adequate to enhance some parameters of cat immune function.

### 3.2. Vitamin K

This fat-soluble vitamin is a component of the vitamin K family. There are two types of natural vitamin K: vitamin K1 and vitamin K2 (45). One of these is more specific for hepatic activating blood clotting factors, while the other one, vitamin K2, is used to activate various extra-hepatic proteins which are more dependent on vitamin K2 than K1. The most common form of vitamin K2 in the human diet is menaquinone-4 and menaquinone-7 (MK-4 and MK-7) (45–48). The effects of vitamin K on the immune response have been shown to be beneficial in various diseases, including inflammatory diseases and cancer (49). During the fermentation process, microorganisms such as Gram-positive bacteria produce a type of compound known as menaquinone, which is actually vitamin K2. This compound is referred to as menaquinone-n due to its 5-carbon-unsaturated carbon chain (50). Another K-family compound is K3, which is synthetically produced without a side chain (51).

**FIGURE 2:**
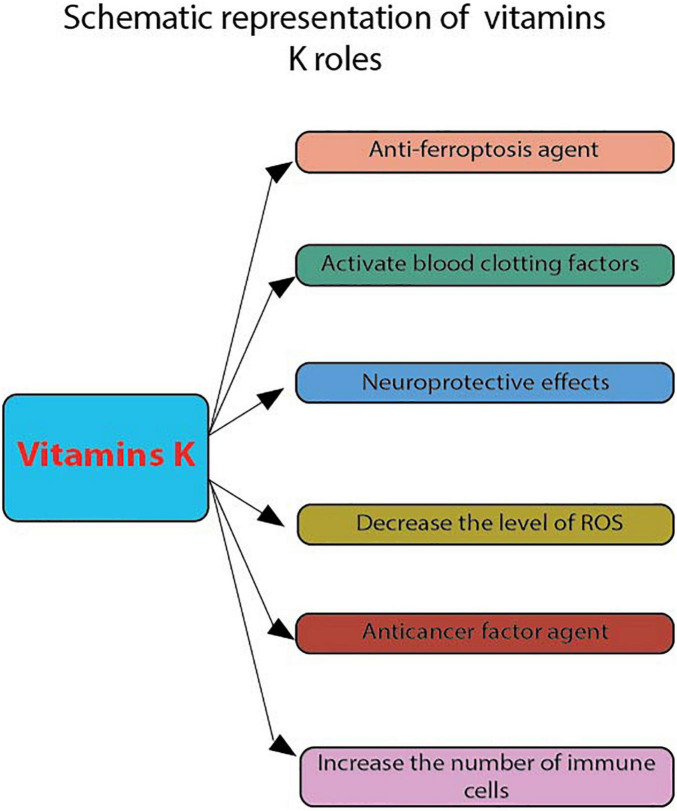
Schematic representation of vitamins K roles. These were evaluated by flow cytometry: anti-ferroptosis agent, activate blood clotting factors, neuroprotective effects, decrease the level of ROS, anticancer factor agent and increase the number of apoptotic cells.

In the nervous system, vitamin K plays a vital role in regulating the activity of certain enzymes involved in sphingolipid metabolism and synthesis. This substance is known to play a role in various cellular events, such as the formation of new cells and the maintenance of cellular communication (52). Studies show that to gain a more substantial anti-ferroptosis effect, fully reduced forms of vitamin K, which include phylloquinone and menaquinone, confer this benefit (Figure 2). The conventional function of this group of naphthoquinones, which is linked to blood clotting, is that it acts as a factor for γ-glutamyl carboxylase (Figure 2). One of the most important factors contributing to the efficient reduction of vitamin K to its hydroquinone form is the presence of ferroptosis suppressor protein 1 (FSP1). This is a NAD(P)H-ubiquinone reductase that is a component of the anti-ferroptosis process (53–55) (Table 1). In this respect, the analysis was conducted through flow cytometry, using BODIPY 581/591 C11 staining. Pfa1 cells were analyzed using a flow cytometer (CytoFLEX and CytExpert 2.4, Beckman Coulter) with a 488-nm laser paired with a 530/30 nm bandpass filter (56). Moreover, while the effects of 6-hydroxydopamine (6-OHDA) on the maintenance of cell viability were not significant, it was observed that the administration of vitamin K2 can protect the growth and maintenance of pheochromocytoma Cell Line 12 (PC12) cells in the Parkinson’s disease. The effects of vitamin K2 on the development and maintenance of PC12 cells have been evaluated using various methods. For instance, the number of apoptotic cells was evaluated using PI staining and flow cytometry (22). The levels of glutathione (GSH), cell viability, and apoptosis were increased in the presence of vitamin K2. However, the levels of reactive oxygen species (ROS) and bcl-2 like protein 4 (Bax) were significantly decreased in the presence of 6-OHDA (Figure 2). In one study, Sada et al. investigated the effects of vitamin K2 on the maintenance of cell viability. They found that it inhibited the activation of the apoptosis-inducing factor in the cell (24). The GATA-binding factor 1 (erythroid transcription factor) mediates cell apoptosis by activating the protein anti-B-cell lymphoma-extra-large (Bcl-XL), a factor that contributes to hematopoietic development.

**TABLE 1:** Vitamins effects evaluated by flow cytometry.

Vitamin name	Investigated model	Pathology/ Biological conditions	Utilized flow markers and flow cytometers	Observed biological effects	Source
Vitamins E	Cell line MDAMB-231-GFP	Human breast cancer cells on mice	Annexin V-PE assay, FACSCalibur flow cytometry (apoptosis analysis)	Anticancer effects via anti-inflammatory mechanisms in some forms	(9, 10)
	Mononuclear cells from the whole blood	Humans exposed to ELF magnetic and electric fields	Annexin V FITC Apoptosis Detection Kit (Sigma-Aldrich), FACS calibur flow cytometry (apoptosis analysis)	Can reduce the oxidative damage caused by ELF magnetic field exposure	(38)
	OSCC cell line ORL-48 and human epidermal keratinocytes (HEK)	Female patient with gum tumor	FragEL DNA fragmentation detection kit, FITC (fluorescein isothiocyanate) Annexin V, flow cytometry system FACscanto II (cytological and apoptosis analyses)	Potential for application in the treatment of oral cancer as a powerful antitumor agent	(39)
	Erythroleukemia cell lines, HEL and OCIM-1, MCF-7 cells, CRL-1740 cells	Breast and prostate cancer cells	Thymidine analogs - [3H] thymidine from amersham (arlington heights, IL) (proliferation analysis)	May prevent the formation of cancer cells as well as smooth muscle cells	(40)
	*Non-murine animal studies* Peripheral blood mononuclear cells	Healthy dogs with a specific diet	CFSE fluorescence FACScan flow cytometer (proliferation analysis)	High plasma levels of vitamin E appear to inhibit the suppression of the lymphoproliferative response	(41)
	T-helper cells, clone: vpg34; cytotoxic T cells, clone: vpg9; clone: CA2.1D6; monocytes, clone: ÜK4	Vaccinated cats with a specific diet	CD4+, CD8+, CD14+ and B cell surface markers, FACSCalibur flow cytometer (immunophenotyping)	Beneficial effects on immune function	(44)
Vitamins K	PC12 cell lines	*In vitro* model of Parkinson’s disease	Propidium Iodide (PI) staining and flow cytometry, EPICS ALTRAII flow cytometer (Beckman Coulter, Brea, CA, USA) (apoptosis analysis)	Pretreatment with K2 can prevent oxidative stress and stop apoptosis in dopaminergic cells, which may slow the progression of Parkinson’s disease	(22)
	Erythropoietin-dependent erythroleukemia cell line AS-E2	Human patients with lymphoma and myeloma in remission	CD11b and CD14, Annexin V-FITC apoptosis detection kit, FACSCalibur flow cytometer (immunophenotyping and apoptosis analyses)	K2 inhibits the activation of the apoptosis-inducing factor in the cell and contribute to hematopoietic development	(24)
	PLC/PRF/5	Human hepatocellular carcinoma cells in vitro and in vivo	Propidium iodide-staining (PI), CycleTest Plus DNA, Reagent Kit (Becton-Dickinson, San Jose, CA, USA), FACScan flow cytometer (EPICS XL, Beckman Coulter; Miami, FL, USA) (cell cycle analysis)	K2 and K3 may be effective treatments for human hepatocellular carcinoma	(21)
	B lymphoma cell line, CTB-1	Human patients with myelodysplastic syndrome	CD33, CD34, CD13, CD14, CD3 mAbs, CD34, CD45, and CD20 mAbs, EPICS XL2 flow cytometer (apoptosis analysis)	K2 may be used for treatment of patients with myelodysplastic syndrome in blastic transformation	(25)
	Peripheral blood mononuclear cells	Healthy adult humans	CFSE dye dilution of CD3 positive, AccuriC6 flow cytometer (proliferation analysis)	Vitamin K2 has immunomodulatory activities, can suppress the proliferation of T-cells in post-menopausal osteoporosis women	(58)
Vitamin B5 (pantothenic acid)	CHO–K1	Chinese hamster ovary cells	BFP2, and GFP marker, CyAn ADP flow cytometer, gallios flow cytometer (cell biology analysis)	Can enhance cell fitness and therapeutic protein production, and alter the lipid metabolism	(72)
	Th1 and Th17 cells	H37Rv-infected mice	FITC-anti-F4/80, APC-anti-CD80, PE-Cy7-anti-CD86, PE-antiMHC-II, PE-anti-CD11b, FITC-anti-Gr-1, APC-Cy7-anti-CD3, PerCP-Cyanine5.5-anti-CD4, APC-anti-CD8a, PE-anti-IFN-γ, and FITC-anti-IL-17, BD LSRFortessa X-20 flow cytometer (immunophenotyping)	Can significantly inhibit the growth of *Mycobacterium tuberculosis* through regulation of innate and adaptive immunity	(73)
	RAW 264.7 cells and BMMs	Female mice	Annexin V/PI apoptosis detection Kit, BD accuri C6 flow cytometer (apoptosis analysis)	Therapeutic potential in prevention of bone loss related disorders	(77)
Vitamins B6 (pyridoxine, pyridoxal, pyridoxamine)	A549, LA-4, HeLa, HepG2, 16HBE	Adenocarcinoma human alveolar basal epithelial cells, mouse lung adenoma cells, human cervix carcinoma cells, human liver hepatocellular carcinoma cells, human bronchial epithelial cells	Green fluorescent protein (PEA/tGFP) polyplexes and polyethyleneimine/t, green fluorescent protein (PEI25k/tGFP) polyplexes with the VBPEA/GFP polyplexes, FACS calibrator system (transfection cell percent analysis)	VB6-coupled poly (ester amine) (VBPEA) can be a promising anticancer therapeutic entity	(92)
	B16 and F10 cells	Mouse melanoma	CD25, ICOS, GzmB, CD44, CD62L near-IR dead cell stain Kit, FACSCanto II flow cytometer (immunophenotyping)	Targeting B6 metabolism may be a useful immunodulatory tactic to enhance anticancer immunotherapy	(93)
Vitamin B9 (folic acid)	Lymphocytes from the lamina propria	Female mice	Anti-CD16/32 antibodies, fluorescent antibodies specific for CD4, ICOS, and GITR, FACSCantoII flow cytometer and FACSAria systems (immunophenotyping)	Vitamin B9 can create an immunological network that aids in the maintenance of treg cells, particularly in the small intestine	(18)
	Cerebral cortex cells	2 day old rats	Fluorescence staining with Hoechst 33342, staining with AnnexinV-FITC and propidium iodide, BD FACSVerse flow cytometer (apoptosis analysis)	Folic acid can reduce oxidative stress, prevent telomeric DNA oxidative damage and attrition	(103)

CD4+, CD8+, CD14+, co-receptor for T-cell receptor; ELF, extremely low frequency, FITC, Fluorescein isothiocyanate; PI, propidium iodide; EPIC, Estimating the Proportions of Immune and Cancer cells; MHC, major histocompatibility complex; CD11b, integrin αM (cluster of differentiation 11b); CD14, cluster of differentiation 14; CD3, cluster of differentiation 3; CD33, transmembrane receptor expressed on cells of myeloid lineage; CD34, phosphoglycoprotein protein; CD45, a type I transmembrane protein; CD20 mAbs, cd20 monoclonal antibodies; CD3mAbs, CD3 monoclonal antibodies; GITR, Glucocorticoid-induced TNFR-related protein; ICOS, inducible T cell co-stimulator.

Certain analyses were performed through flow cytometry, such as cell differentiation (24). In another study, Gehrmann et al. (57) found that vitamin K2 can reduce the production of reactive nitrogen species (RNS) through the NFkB-iNOS pathway. Due to its low toxicity, vitamin K has been considered a potential cancer-fighting agent (Figure 2). However, there are only a few studies that have investigated the effects of this substance on the growth and maintenance of cancer cells (21). The results of flow cytometry analysis revealed that the presence of vitamin K1 and vitamin K2 did not induce cell cycle arrest or apoptosis in PCL/PRF/5 cells (21). Also, the presence of vitamin K2 in patients with different types of leukemia can have an apoptosis-inducing effect on their newly isolated cells. Multi-color flow cytometry was the technique used (25). Moreover, it was also reported that vitamin K2 can suppress the proliferation of T-cells in osteoporotic patients. This study was conducted using flow cytometry based on carboxyfluorescein succinimidyl ester (CSFE) dye dilution. The T-cell proliferation was then determined by CFSE dye dilution of CD3 positive using an AccuriC6 flow cytometer (58) (Table 1). As such, poor bone development and an increased risk of cardiovascular disease are some of the effects of vitamin K deficiency. The rare occurrence of vitamin K toxicity is attributed to the presence of a component called menadione, which is not used in humans. When this happens, it can cause various symptoms such as jaundice and hemolytic anemia. The toxic effects of menadione can be triggered by the increased levels of oxygen that the liver takes in. This increases lipid peroxidation, which then leads to cell damage and death (59).

## 4. Water-soluble vitamins

When they enter the body, water-soluble vitamins dissolve in water. This is the reason that the human body does not store the excess but possibly aims at their new use. There are nine types of water-soluble vitamins. One is represented by the C vitamin and the rest of the vitamins belong to the B group. The deficiency of any type of these vitamins is associated with clinical abnormalities, that vary from anemia to neurological disorders (60, 61). Therefore, water-soluble vitamins are essential for the normal functioning of cells, their proliferation, and development. In fact, their health concentration which is responsible for maintaining the previously exposed functions depends somewhat on their normal absorption from the intestine (61). Even if all B vitamins are soluble in water, they are very different structurally and functionally. Normally, all these vitamins are found in sources of animal and plant origin. In addition, there are many foods that are fortified with such vitamins, especially due to deficiencies having been reported (62).

### 4.1. B5 vitamin (pantothenic acid)

**FIGURE 3:**
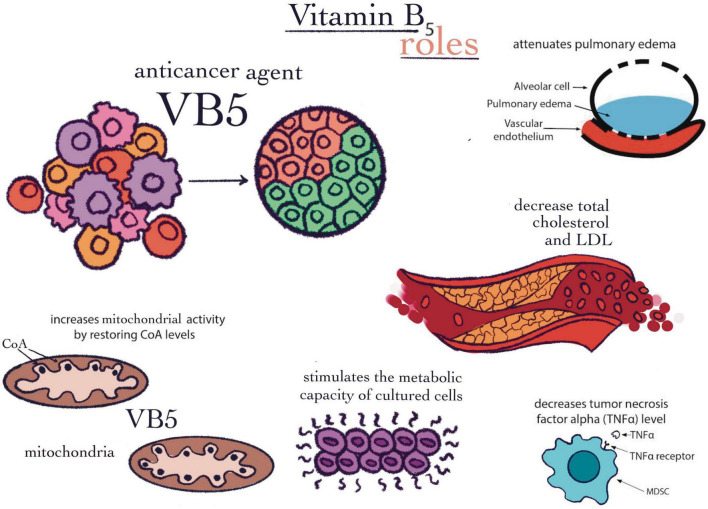
Vitamin B5 roles. The following effects were presented: Anticancer agent, attenuates pulmonary edema, decrease total cholesterol and LDL, increases mitochondrial activity by restoring CoA levels, stimulates the metabolic capacity of cultured cells, and decreases tumor necrotic factor-alpha (TNF-α) level.

Vitamin B5 is water soluble and can be synthesized from the diet and intestinal microflora (63). It is found in most foods and is considered the precursor of CoA, as it participates in many metabolic reactions, especially in energy-producing reactions such as fat and carbohydrate metabolism, and less in protein metabolism (64). As a precursor of CoA and acyl carrier proteins, vitamin B5 increases mitochondrial activity by restoring CoA levels (65) (Figure 3). Therefore, coenzymes containing pantothenic acid are involved in energy reactions and are also specific to lipid metabolism (66). According to a recent study, the differentiation of some cytotoxic cells into cells that produce interleukin, e.g., IL-22, is stimulated by vitamin B5 and CoA. They are, in fact, CD8 + Tc22 cells. By exogenous administration of CoA to reprogrammed T-cells, they can adopt the CD8 + Tc22 phenotype. Tc22 cells that produce IL-22 are considered to be the cells with the strongest antitumor activity from CD8 + T-cell subsets. In mice, they were observed to have a treatment-enhancing action through programmed death-ligand 1 (PD-L1) antibodies against cancer (67).

According to current literature, the increased circulating levels of B5 should be correlated with a good state of health that is reflected in effective immune responses (63, 68). Moreover, by increasing the level of SOD in the lungs and inhibiting the accumulation of neutrophils, B5 derivatives significantly attenuate pulmonary edema (69) (Figure 3). In addition, in patients with endometriosis, B5 derivatives decrease tumor necrosis factor alpha (TNF-α) levels and reduce oxidative stress (70) (Figure 3). Also, vitamin B5, alongside other vitamins, is capable of improving mental health (71). In a study by Pourcel et al. (72) it was demonstrated by flow cytometry that cultured cells have an improved metabolic capacity in the presence of vitamin B5 compared to unexposed cells (Table 1). The percentage of fluorescent cells was determined using the CyAn ADP flow cytometer (Beckman Coulter), as well as the fluorescence intensity of GFP-positive cells. In short, the protein production and cell homeostasis state may be stimulated by the lack of vitamin B5 (72). In the case of tuberculosis, it has been demonstrated that vitamin B5 has antibacterial effects. *In vivo*, the growth of *Mycobacterium tuberculosis* was suppressed by the administration of vitamin B5. The mechanisms involved belong to innate, as well as adaptive immunity. It was discovered that the stimulation of TNF-α and IL-6 expression simultaneously with the intensification of signal molecules such as ERK and AKT can be achieved by vitamin B5. Initially, neutrophils are harvested and washed. After they are stained on ice for 30 min with mapping cell population (mAp) mixtures that were previously fluorescently conjugated or isotopes that matched the control (73). The technique used was also flow cytometry in the assessment of the phagocyte capacity of cells and could be performed after pretreatment with vitamin B5 and then with the help of fluorescent Texas-Red-labeled MTB H37Rv (74). Another area where vitamin B5 is quite used as treatment is the management of dyslipidemia. It is responsible for lowering total cholesterol and LDL (75) (Figure 3). Interestingly, there are studies that show that in pregnant or breastfeeding women, the administration of B5 in large doses may cause episodes of diarrhea or even intestinal pain (76). When there is a deficit of estrogens, a diet rich in pantothenic acid has an improving effect on the loss of bone mass. This effect was evaluated with the help of flow cytometry, which mainly analyzed cell apoptosis with several concentrations of pantothenic acid used in this study. The effects of different concentrations of pantothenic acid on cell viability were followed as Annexin V/PI Apoptosis Detection Kit (KeyGEN, Biotech) was used to obtain these results (77) (Table 1).

### 4.2. B6 vitamin

The vitamin includes a group of 6 chemical compounds that have the pyridine ring in common. VB6 is considered an essential micronutrient due to the incapacity of the human body and animals to synthetize it (78, 79). In contrast, VB6 can be produced by bacteria, fungi and plants. In humans, it can be assimilated from food (80). A lack of dietary variety is usually the main cause of B6 deficiency, both for animals (81) and for humans (82). Cereals, poultry, beef, and potatoes are the main sources of VB6 (82, 83). Given the importance of this vitamin for the health of all living organisms, research nowadays is testing genetic engineering techniques to create commercial crops with a greater ratio of VB6 (82, 84, 85).

**FIGURE 4:**
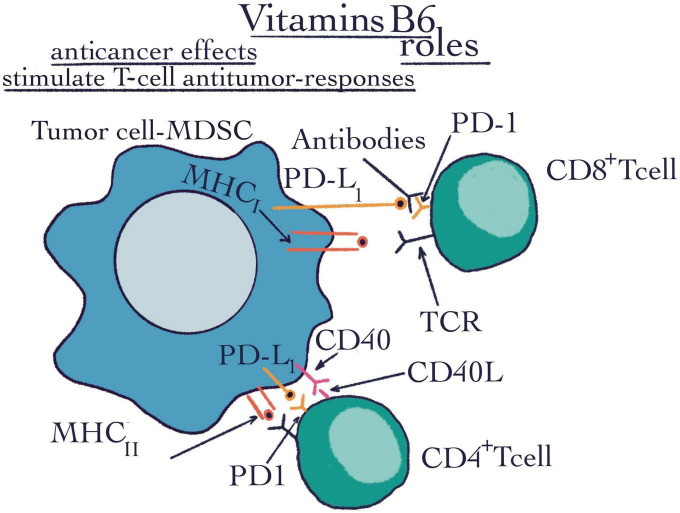
Vitamins B6 roles. The flow cytometry technique highlighted the roles of vitamins B6 such as anti-cancer effects and stimulating T-cell anti-tumor responses. CD4+ T-cell, CD4+ positive T-cell is a subtype of lymphocyte, they are MHCII restricted and are important mediators of adaptive immunity; CD8+ T-cell, CD8+ positive T-cell is a critical subpopulation of MHCI restricted T-cell; MHCI, major histocompatibility complex class I; MHCII, major histocompatibility complex class II; PD-1, programmed cell death protein 1; PDL-1, programmed cell death ligand 1; CD40, a cluster of differentiation 40; CD40-L, a cluster of differentiation 40 ligand; R, receptor; TCR, tumor cell receptor; MDSC, myeloid-derived suppressor cell.

As a critical co-factor, it is involved in more than 150 metabolic reactions and in cellular signaling. In addition, VB6 has the ability to decrease the final products of glycation (78, 86, 87). It was first discovered as a treatment of dermatitis in rats (88), then, with the increasingly active research of this micronutrient, it was demonstrated that several diseases like, diabetes, pneumonia, atherosclerosis, heart disease or even the resistance to COVID-19 can be connected with B6 deficiency (87, 89). Also, Field et al. revealed that supplemental VB6 increased surround suppression of visual contrast detection, decreased self-reported anxiety, and showed a trend toward depression reduction (90). Moreover, it seems that VB6 may be regarded as possible anti-tumorigenic food resource (91). Much of the research on the possible anticancer effects of VB6 is carried out by flow cytometry of utmost importance in the study of cancer (Figure 4). Using this technique, Pandey et al. (92) determined the effectiveness of transfection and poly (ester amine) gene transporter-coupled VB6 membrane transport in cancer cells. The technique was performed to see the efficiency expressed in percentages of the transfection with regard to the phosphoprotein enriched in astrocytes/t green fluorescent protein (PEA/tGFP) polyplexes and polyethyleneimine/t green fluorescent protein (PEI25k/tGFP) polyplexes with the VBPEA/GFP polyplexes (1 μg) in A549 cells. Harvested transfected cells were washed with 1× PBS. Subsequently, in order to determine the transfection percentage, the cells expressing GFP were again labeled by FACS (1 × PBS) (92). Also, flow cytometry was used to highlight that T-cell anti-tumor responses are determined by VB6 metabolism (Figure 4). Therefore, fluorochrome-conjugated monoclonal antibodies were used to stain the surface, cytoplasm, and/or nuclear components of single cell suspensions after the use of Near-IR Dead Cell Stain Kit (93) (Table 1).

### 4.3. Vitamin B9 (folic acid)

**FIGURE 5:**
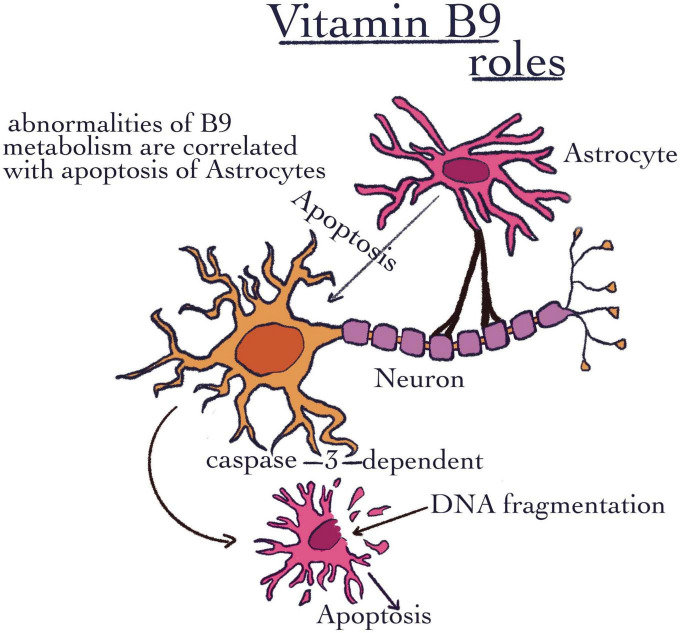
Vitamin B9 roles. The image shows the importance of VB9, which was highlighted through abnormalities of B9 metabolism that are correlated with the apoptosis of astrocytes.

Similar to B5, this vitamin is water-soluble. As it was previously mentioned, vitamin B9 is necessary to maintain regulatory T-cells (18). This vitamin may be uptaken from intestinal bacteria as well as from the diet (94). It was demonstrated that folate has two different places of absorption. The dietary form can be absorbed in the jejunum, while in the colon, there are bacteria that can synthesize folates. Thus, the bacteria from the colon represent a potential endogenous source of folates (95). Its role in the regulation of Treg cells was also evaluated by flow cytometry. Both the sorting and the actual method of flow cytometry were carried out by the following methods of Gohda et al. (96, 97) (Table 1). The first technique followed preincubation with a 10 g/ml anti-CD16/32 Ab mixture (BD Biosciences). However, in the CD3 T-cell fraction, this treatment increased the CD8 population and it is also responsible for stimulating the proliferation of nerve stem cells in the brain, which has a beneficial impact on neurodegenerative diseases (98). Moreover, vitamin B9 is capable to maintain and even proliferate the Schwan myelin-producing cells (99). Also, abnormalities of folic acid metabolism in the brain are correlated with increased apoptosis of astrocytes (Figure 5). In this respect, an annexin solution was used to stain the harvested cells. The procedure took 10–15 min, after which the cells were resuspended in the binding buffer. The detection of apoptotic cells was performed using a FACS-Calibur cytometer (BD Bioscience) (100). Unfortunately, it has been shown that this phenomenon can become favorable for the apparition of neurodegenerative disorders such as Alzheimer’s disease (101, 102). The decrease of apoptosis capacity is possible due to the folic acid ability to prevent oxidative stress. To this end, flow cytometry and Hoechst 33342 fluorescence staining were used to measure astrocyte apoptosis. The apoptotic character was determined by the condensation and fragmentation of the chromatin in the nucleus (103) (Table 1). Saxena et al. (104) observed a link between maternal folic acid levels and Autism Spectrum Disorders (ASD), due to its role as a precursor of S-adenosyl-methionine (SAM). SAM is considered the folate donor in the DNA methylation process. It has a role in maintaining amino acid homeostasis, and in the fight against reactive oxygen species, all due to its role as a cofactor in a multitude of one-carbon transfer reactions (105). In contrast, there are also some data that found a correlation between the over expression of folic acid receptors and several types of cancer, such as kidney, ovarian, and breast cancer (106–108). There is a trend nowadays to fortify certain foods with vitamin B. The negative effects of excess are not well known (109). Excess folic acid is converted in the liver under the action of dihydrofolate reductase into tetrahydrofolate. When folic acid is in excess, the enzyme is saturated, and folic acid can no longer be reduced. Thus, an excess of unreduced folic acid results, which can be considered a possible mechanism for carcinogenesis (109, 110). Moreover, excess folate can have repercussions on replication and ultimately on survival of cancer cells. In addition, when enzymes are saturated, their efficiency is reduced. Due to it, the metabolism of nutrients can be changed. Finally, the risk of some diseases can be increased (111). In contrast, an insufficient quantity of folic acid can cause DNA hypomethylation (112). Cytosine methylation in the genome is linked to the expression of certain genes (113). If the hypomethylation is specific to the promoter regions of some genes, then the control of transcription and distinctly the suppression of some genes can be modified. Thus, folic acid deficiency, which results in hypomethylation, may result in the induction of some proto-oncogenes. These are known for their role in tumorigenesis (114). Inactivation of promoter regions of suppressor genes can be caused by hypermethylation.

## 5. Conclusion

It has been clearly established that vitamins have a role in immunomodulation. Vitamin E is used worldwide as a food supplement to reduce oxidative stress that leads to pathological conditions. Also, it is used as cholesterol-lowering agent and inhibitor of hepatic steatosis. Additionally, K vitamins can be responsible for the decrease of pro-inflammatory cytokines, with a special impact on the body’s fight against infections. Moreover, they have an antioxidant effect, as K2 increases the levels of glutathione (GSH), cell viability, and apoptosis. Vitamin B5 is involved in the differentiation of some cytotoxic cells into CD8 + Tc22 cells that produce interleukin, e.g., IL-22, while in cancer treatment, it exhibits a treatment-enhancing action. Similarly, several diseases such as, diabetes, pneumonia, atherosclerosis, heart disease, or even the resistance to COVID-19 can relate to B6 deficiency. Once T-cells differentiate from naïve T-cells express on their surface (FR4) and need B9 to survive. As a cofactor for a multitude of one-carbon transfer reactions, it is involved in amino acid homeostasis and in the fight against reactive oxygen species. The excess of folic acids is associated with the risk of some diseases, including cancer. Since the importance of vitamins in immunity is crucial, the techniques evaluating these properties are also essential. In this respect, flow cytometry is revealed as a good method to evaluate the immunomodulatory effects of certain vitamins.

## Author contributions

CM and AO: conceptualization, investigation, and writing original draft preparation. CM, IB, MM, BP, MP, LM, OP, and AO: investigations, collected the data, data interpretation, and preparation of the manuscript. LM, IB, MM, BP, MP, OP, and AO: validation, writing review, and editing. CM: supervised the work. All authors contributed to the article and approved the submitted version.
